# Valvular Aortic Constrictive Disease

**Published:** 1857-03

**Authors:** F. B. Norcom


ARTICLE II.—'*Valvular Aortic Constrictive Disease.*' By F. B. NORCOM, M.D.

*Ward* 9 *and* 10.—Thos. Smith; aged forty-two years; Irishman; unmarried; a stone-quarryman by occupation; and admitted to Bellevue Hospital, Sept. 1st, 1854.

His father died of inflammation of the lung; yet there exists no hereditary tendency to disease in family or its branches, ten children having reached maturity, with two exceptions, in the enjoyment of vigorous health.

The patient has been a man of intemperate habits, and always lived freely; possessing good health until ten years ago, when he contracted syphilis. He denies the existence of a primary sore, but states that his attention was first directed to his condition by the occurrence of buboes in both groins, which suppurated freely, and were not fully cicatrized until eighteen months after the date of commencement.

Eight months after this period, he began to experience osteocopic pains, involving the knee-joints and long bones of the lower extremities; paroxysmal in character, and increasing in severity during the night. Simultaneously, an inflammation of the eye, attended with photopsia, pain, and perversion of vision. These specific accidents lasted some three months, and he then remained in fair condition during an interspace of four or five years, when he was attacked by the 'fever and ague' of the tertian type, from which he suffered more or less for one year. Has been salivated twice during the treatment for his venereal disorder. Never had rheumatism.

This sufferer dates his present trouble from a year last January. Was returning from his work one evening, after having been engaged in heavy lifting, and observed that his breathing became suddenly short and difficult. This now increased so rapidly, that he was obliged to stop and rest himself, and was assisted home finally by his comrades. He recovered, however, to return to his business the next day, but has never felt the same man since, consequently engaged in less laborious occupation; yet was subject from time to time to fits of dyspnœa, which would be readily excited by moral causes, exposure to extremes of weather, or unusual exertion.

About the middle of August was forced to give up all work, swelling of his feet, palpitations, constant dyspnœa with cough, insomnia, and capriciousness of appetite having materially added to his sufferings. On admission, he not only complained of the above symptoms, but of a sense of throbbing in the neck and left breast, most painfully increased by the recumbent posture at night. After remaining in the wards a month, found himself so much improved that he requested a dismissal, proposing to resume his vocation; but after a short time was forced to desist, and re-entered the hospital two days from his exit, where he has remained up to his decease.

During the past nine months he has grown gradually worse, the intervals between his attacks (two or three weeks) being much shorter, occurring in as many days and generally at night. These asthmatic paroxysms are associated with pallor of the face, profuse perspiration, rapid and laborious respiration, the pulse running over one hundred, hard and irregular, an intense choking sensation, and severe ringing cough, free from expectoration. They continue two hours or more, and are accompanied and followed by violent cephalalgia, with dimness of vision, and inability to lie upon the left side. No turgesence of the veins of the neck; no dysphagia; and during the intervals the cough is not stridulous. The radial pulses are synchronous; the left weaker than the right; and present the locomotive character of aortic regurgitation in a marked degree. Bowels regular, and urine healthy.

*Physical Examination.*—Point of apex of heart indeterminate; no thrill or impulse upon palpitation; and in the second intercostal space, next to the sternum, is a pulsation synchronous with the action of the heart. No abnormal extent of dulness upon percussion. Over the cardiac region is heard a double *bruit*, systolic and diastolic, loudest at apex. The same also heard in still greater intensity three inches to right of median line, in the second intercostal space, a little below the pulsating point above referred to, and clearly transmitted to carotid and subclavian arteries; the latter giving a powerful pulsatile movement. Aorta beats with abnormal force in jugular fossa, although not elevated above upper edge of sternum.

No material change occurred in the physical signs or cardiac symptoms; towards the decline of life, however, his troubles were greatly aggravated by the increased frequency of these paroxysms of cardiac asthma, and the enormous œdema of his lower extremities. Expired on the 15th of August.

*Autopsy.*—The examination revealed the existence of constrictive disease of the semi-lunar aortic valves, which rendered them insufficient. No aneurism, and but slight dilitation of the aorta, which presented numerous patches of artheroma. Heart somewhat hypertrophied. Mitral valves healthy. Thoracic and abdominal viscera conjested.

A careful review of this case and the assemblage of symptoms will exhibit—despite the perfection to which physical examination has been brought—the difficulties to be encountered in forming a correct diagnosis of the various thoracic diseases presented to our consideration, and which we are apt not only to undervalue, but, in the plenitude of our experience and success, to pronounce upon each disease with a certainty, which the results of a microscopic inspection will often fail to justify.

This patient was subjected to repeated examinations, conducted by reliable diagnosticians; and the opinions formed were invariably different—the weight, however, favoring sacular dilitation, while a few maintained the existence of regurgitative disease. The laborious occupation of the man, the suddeness of the attack, the blood deterioration from specific poison, and many of the physical signs, taken in connection with the sense of throbbing and the choking sensation—due to mechanical pressure, and generally pathognomonic of aneurismal formation—will approve the former view; while, again, the character of the pulses, the absence of thrill, want of dulness, no signs of external prominence, will offer a sufficiently valid excuse for the latter. Percussion gave no abnormal extent of dulness; but this fact is frequently a source of error, dulness not always being co-extensive with the dimensions of sacular dilitation.

The situation of the sac, its more or less globular form, a limited portion only reaching the chest walls, would preclude this sign *alone* from giving much importance to the examination.

And we may here deduce a practical result from this fact, that such sacs are larger than the results of percussion would indicate,* offering an explanation of the grave subjective symptoms frequently witnessed in these cases, even though the physical signs failed to point out their causation. The pulsating point referred to, and the organic conditions of arterial murmur, supposed referable to change of form in the vessel, and to change of internal surface, are presumptive of dilitation: for, if the arterial murmurs are audible at any point of the arch, and of different pitch, or greater intensity, and harsher quality than a synchronous murmur at the aortic base, it may be inferred that the cause of intensification resides in the vessel itself.† In aneurism of the aorta, œdema of the lower extremities seldom occurs, a fact that may have its diagnostic application, yet, in the example before us, will be antagonized by another of equal importance, that constrictive disease may exist also to a most remarkable extent without producing systemic stagnation. This immunity, however, will only hold good, in either case, where the capacity of the ventricles and patency of the tricuspid orifice remain normal or sufficient, yet, should the blood become spanaemic, then anasarca will occur independently of these pathological changes.

Atheroma, unless accumulated in large quantities, will not produce murmur; and occurring, as it does, after the middle period of life, is perhaps the most frequent cause of arterial or valvular disease.‡ Another frequent cause of these organic changes, and one often overlooked, takes its origin from syphilitic infection. In our examinations of those affected with heart disease, one of our first questions, is, if he ever suffered from rheumatism; if not, we then proceed to a rigorous investigation of other organs, in order to obtain some satisfactory clue to this pathological condition. The fact of his ever having contracted pox, is gleaned from the general examination of the patient, and not with a view of its bearing upon this particular point, or from any just appreciation of its being one of the cer-

* Walshe on the Heart and Lungs.
† Walshe, Op. Cit. p. 231.
‡ Swett on Diseases of the Chest.

tain and efficient causes producing cardiac lesion. As an agent deteriorating the blood, there exists few stronger, and its continual presence — a total and absolute elimination from the system, being highly problematical—must, as a consequence, induce variations in the nutritive or vital processes of the economy.

It is well understood, that, for the healthy nutrition of the body, certain conditions are necessary: one is a *right* condition of the blood—in other words, a certain adaptation between this fluid and the tissues, and even the several portions of each tissue; another recognizes a healthy state of the part as essential. Now, each and every condition must prove defective where the blood is saturated, as it were, with some noxious agent, continually multiplying itself, giving evidence of its existence by morbid manifestations, and inducing a specific cachexia, in every sense adverse to a healthy performance of the various functions. It may not be uninteresting to observe here, that syphilis took its starting point from a *bubon d' emblee*, and running on to suppuration was followed by constitutional accidents.

The experimental researches of M. Ricord, however, leave no room to doubt that a primary sore must have existed in this man (though it probably escaped his notice), and that the formation of pus in the glands, if such the case, was ascribable to some vicious or pyogenic (if we may so term it) habit of the system.

